# Renin Activity in Heart Failure with Reduced Systolic Function—New Insights

**DOI:** 10.3390/ijms20133182

**Published:** 2019-06-28

**Authors:** Ryan D. Sullivan, Radhika M. Mehta, Ranjana Tripathi, Guy L. Reed, Inna P. Gladysheva

**Affiliations:** Department of Internal Medicine, University of Arizona College of Medicine—Phoenix, Phoenix, AZ 85004, USA

**Keywords:** heart failure, plasma renin activity, renin–angiotensin–aldosterone system, renin, prorenin, (pro)renin receptor, reduced systolic function, dilated cardiomyopathy, direct renin inhibitor, aldosterone

## Abstract

Regardless of the cause, symptomatic heart failure (HF) with reduced ejection fraction (rEF) is characterized by pathological activation of the renin–angiotensin–aldosterone system (RAAS) with sodium retention and extracellular fluid expansion (edema). Here, we review the role of active renin, a crucial, upstream enzymatic regulator of the RAAS, as a prognostic and diagnostic plasma biomarker of heart failure with reduced ejection fraction (HFrEF) progression; we also discuss its potential as a pharmacological bio-target in HF therapy. Clinical and experimental studies indicate that plasma renin activity is elevated with symptomatic HFrEF with edema in patients, as well as in companion animals and experimental models of HF. Plasma renin activity levels are also reported to be elevated in patients and animals with rEF before the development of symptomatic HF. Modulation of renin activity in experimental HF significantly reduces edema formation and the progression of systolic dysfunction and improves survival. Thus, specific assessment and targeting of elevated renin activity may enhance diagnostic and therapeutic precision to improve outcomes in appropriate patients with HFrEF.

## 1. Introduction

Symptomatic heart failure (HF) with reduced ejection fraction (rEF) affects millions and is the most common reason for heart transplantation [1,2,3]. The prevalence of symptomatic HF will increase 46% from 2012 to 2030 [4]. Dilated cardiomyopathy (DCM) due to nonischemic or ischemic causes is characterized by progressive heart enlargement with rEF, which is a major risk factor for the development of symptomatic heart failure with reduced ejection fraction (HFrEF) [2,3,5,6].

The American College of Cardiology/American Heart Association (ACC/AHA) staging system suggests that HFrEF predictably progresses though four stages, from A to D. Patients at risk for DCM have Stage A HF and usually progress to Stage B, where there is a decline in systolic function; both Stages A and B are without symptoms or are presymptomatic. Subsequently patients develop symptomatic HF (Stages C and D), which is associated with fluid retention (edema), breathlessness, fatigue, exercise intolerance, and death [7]. Transition from presymptomatic HF to symptomatic HF is critically important for patients as it leads to a marked decline in the quality of life and is associated with significant morbidity and mortality [4,8].

Regardless of the cause of DCM, the progression from asymptomatic to symptomatic HF is associated with interdependent, neurohormonal alterations of the sympathetic nervous system (SNS), the renin–angiotensin–aldosterone system (RAAS), and the natriuretic peptide (NP) system [9,10,11,12,13]. Neurohormonal activation may initially compensate for impaired cardiac function; however, prolonged activation has deleterious effects on cardiac structure and performance, leading to symptomatic HF associated with edema [8,13,14]. An elevation in plasma norepinephrine signifies SNS activation, which occurs at Stage B in clinical and experimental HF and is strongly associated with progression of systolic dysfunction, congestion, and RAAS activation [15,16]. The NP system acts to counter the SNS-RAAS by promoting diuresis, natriuresis, and vasodilation [11,17,18,19,20,21]. However, the effects of the NP system are attenuated in advanced HF Stages C and D [22,23,24,25]. Activation of RAAS is associated with left ventricular dysfunction, cardiac dilation, sodium and extracellular fluid retention (edema), and cachexia/sarcopenia [26]. Systemic RAAS activation occurs with increased plasma renin activity, which initiates the activation of the primary targets for clinical intervention: angiotensin II (Ang II) and aldosterone [27]. Pharmacological blockade of RAAS has proven to be the mainstream/standard treatment approach for symptomatic HFrEF patients [28,29,30,31,32,33,34,35,36,37]. Standard medical therapies include RAAS blockers: angiotensin converting enzyme (ACE) inhibitors (ACE-I), which block the enzymatic conversion of Angiotensin I (Ang I) to Ang II; Ang II receptor blockers (ARB), which block the binding of Ang II to the Ang II type I receptor (AT1); mineralocorticoid receptor antagonists (MRA), which block effects of aldosterone; and angiotensin receptor/neprilysin (NEP) inhibitors (ARNi) [38,39]. Direct renin inhibitors (DRI) are designed to block enzymatically active renin from triggering a pathological alteration of downstream pathways. However, clinical trials have failed to demonstrate the value of renin activity inhibition for improving outcomes for patients with HF on concurrent RAAS blockers or diuretics [40,41,42]. Complicating the issue is the heterogeneity of patient plasma renin activity levels and their individualized responses to plasma renin activity levels on the background of RAAS blockers or HF progression itself.

Here, we review the role of active renin in HFrEF progression and the potential benefits of renin activity as a diagnostic and/or prognostic plasma biomarker, as well as the potential value of renin inhibitors as a targeted therapy for HFrEF. 

## 2. Pathways of Renin Generation and Function

**Figure 1:**
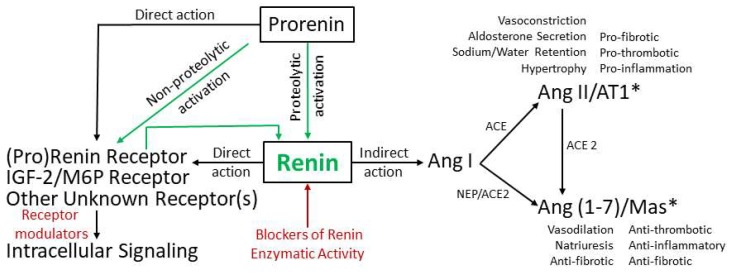
Schematic representation of the potential role of renin activity in the modulation of heart failure with reduced ejection fraction (HFrEF) via direct and indirect effects: Further complicating the physiology of renin is that it has systemic and local effects within the heart. The green arrows represent proteolytic and non-proteolytic conversions of prorenin to enzymatically active renin. Enzymatically active renin has the potential to directly modulate HFrEF progression through binding with receptors such as the (pro)renin receptor and/or IGF-2/M6P receptor. It can also act indirectly through the generation of Angiotensin I (Ang I), which acts through the angiotensin converting enzyme (ACE)-angiotensin II (Ang II)/Ang II type I receptor (AT1) and neprilysin (NEP)/Angiotensin Converting Enzyme 2 (ACE2)-Angiotensin (1–7) (Ang (1–7))/Mas axes or possibly Ang II type 2 receptor (AT2) (*).

Systemic RAAS maintains vasoconstriction, retains sodium and water, increases arterial blood pressure, and increases myocardial contractility, thus increasing the effective circulating blood volume during physiological and pathological conditions including HF [43,44]. Pathologically activated RAAS promotes symptomatic HFrEF [11,45]. The systemic RAAS activation cascade begins with production of prorenin and its conversion/activation to renin (Figure 1), an enzyme that was discovered more than a hundred years ago [46]. Renin activity in plasma increases as HFrEF progresses and is highest with symptomatic HFrEF in patients and animals. The prorenin/renin pair directly and indirectly modulates HFrEF progression. Figure 1 schematically represents a potential role of renin activity in the modulation of HFrEF through direct and indirect actions.

Renin is a monospecific aspartic protease [47] that cleaves glycoprotein angiotensinogen systemically in the circulation or locally at the tissue level to generate angiotensin I (Ang I), which is the key and rate-limiting step in the RAAS activation cascade. Thus, while the concentration of angiotensinogen in plasma is approximately 500–600 pmol, Ang I is present in plasma in at least 10,000-folds lower concentration in the 50- to 100-fmol range and Ang II at approximately half of Ang I [48]. It is important to note that circulation levels of angiotensinogen are in the range of the Michaelis–Menten constant for renin; therefore, small modulations in either renin or angiotensinogen levels may significantly alter downstream outcomes [49]. Ang I is the precursor of the physiologically active peptides, Ang II and Angiotensin (1–7) (Ang (1–7)). These are generated by different enzymes, ACE or ACE 2 and NEP; they act through distinct cell surface receptors with opposing biological effects (e.g., vasoconstriction vs. vasodilatation, etc.). However, how these interdependent effects modulate HFrEF is poorly understood. Ang II binds AT1 in targeted tissues and stimulates vasoconstriction and secretion of the steroid hormone aldosterone, which mediates sodium reabsorption and water retention [50,51]. Ang (1–7) is a vasodilator that works through the Mas receptor and opposes the actions of increasing Ang II [52,53,54,55,56,57,58,59]. Ang II and Ang (1–7) might also execute their actions through the Ang II type 2 receptor (AT2) in tissue-dependent action [57,60,61]. Ang II actions on the AT2 receptor are thought to be beneficial through vasorelaxation, antihypertrophic, and anti-fibrotic effects. However, AT2 receptor effects on the pathophysiology of HFrEF are predominantly reported during AT1 blockades with ARBs), which are associated with increased plasma renin concentration and activity [62,63,64]. Despite the extensive experimental and clinical research, the role of AT2 receptor signaling in the failing myocardium remains controversial [65,66,67,68]. The balance between Ang II and Ang (1–7) arms may be disturbed during HFrEF progression, both at a systemic level (in circulation) and local level (in tissues). Whether pharmacological alteration of pathological renin activity can restore balance to these pathways remains unclear.

In mature mammals, renin is primarily expressed in juxtaglomerular (JG) cells and the collecting duct in the kidney as an enzymatically inactive precursor, prorenin. Prorenin contains a 43-amino-acid prosegment that covers the active cleft of renin to prevent intracellular proteolysis and to provide folding, stability, and extracellular sorting [69,70,71,72]. Mechanisms controlling prorenin expression and prorenin/renin secretion have been comprehensively reviewed [44,73,74,75]. Prorenin/renin expression and secretion are tightly regulated. At the cellular level, they are regulated by calcium ions (inhibitory) [76,77], cyclic adenosine monophosphate (cAMP; stimulatory) [73], and cyclic guanosine monophosphate (cGMP; inhibitory) [78,79,80]. Ang II levels inhibit renin release through a negative feedback loop, which is activated largely by the influence of the pathologic state or by the use of RAAS medications [44,81,82]. At the organ level, they are regulated by two distinct mechanisms: through a renal baroreceptor and by macula densa sodium chloride transport [44,83,84,85]. Hence, prorenin/renin levels in circulation may be altered in response to stimuli like hypertension, sodium ion concentrations, pharmacological agents including ACE-I, ARBs, loop diuretics, and others [44]. 

Prorenin potentially can be activated in two ways: proteolytic or non-proteolytic (without proteolytic cleavage). Proteolytic activation involves the actual removal of the prosegment. It occurs in the vesicular network of juxtaglomerular cells by a specific (still unknown) renal protease and potentially in circulation by the kallikrein/Factor XII network [86,87,88,89,90,91,92,93]. Experiments performed in cathepsin B-deficient mice ruled out this intrarenal enzyme as a prorenin convertase [94]. Non-proteolytic activation requires conformational change in the prorenin structure, leading to the removal of prosegment from the active enzymatic cleft without proteolytic cleavage, which is achieved after prorenin binds to tissue-expressed (pro)renin receptor, (P)RR [95]. Accumulated data suggest that, while expressed, the majority of prorenin (up to 80%) is directly released into circulation, while enzymatically active renin remains stored in the vesicular network awaiting controlled release [44,74,96]. Prorenin circulates in blood at concentrations that are ten times higher than active renin [72]. Thus, renin activity in circulation might be modulated by the alteration of active prorenin/renin secretion, stimulation of prorenin activation in the circulation, or inhibition of enzymatic renin activity in circulation. Direct renin inhibitors (DRI) were designed to pharmacologically block enzymatic renin activity in circulation generated proteolytically as well as non-proteolytically [97]. 

The prorenin/renin network in the kidney is believed to be responsible for activation of RAAS not only systemically in circulation but also locally at the tissue level through prorenin/renin receptor (P)RR-binding mechanisms. (P)RR is a 350-amino-acid protein with a single transmembrane domain, which is widely expressed in various tissues, including the heart and kidney, and acts in a prorenin/renin dependent [95,98,99] and independent fashion [72,100]. After prorenin binds to the (P)RR, non-proteolytically active and fully enzymatically active renin are released back into circulation to trigger systemic RAAS or to work locally at the tissue level to generate Ang I, which converts to the RAAS primary effector peptides, Ang II/Ang (1–7), that are involved in the pathophysiology of cardiac remodeling [57,101,102,103,104,105]. Kidney-derived renin or prorenin required for Ang II generation in the cardiac tissue [43,103,106,107] is taken up from circulation through diffusion in the interstitial space or by the (P)RR or other unknown renin receptor(s) [95,101,102,103,104,108,109,110,111,112]. (P)RR non-proteolytically activates prorenin also acts as a signaling receptor for the prorenin/renin pair to activate multiple intracellular post-receptor cascades [113,114,115]. Thus, in cell models, the interaction of (P)RR with prorenin is reported to be important for activation of the mitogen-regulated protein kinase (MAPK) and extracellular signal-regulated kinases 1 and 2 (Erk1/2), leading to production of profibrotic genes, including transforming growth factor-β1 (TGF-β1), plasminogen activator inhibitor type 1 (PAI-1), fibronectin, and collagen [72,98,99,116,117]. Aside from prorenin/renin-dependent functions, (P)RR is involved in vacuolar-type H^+^-ATPase (V-ATPase), Wnt/β-catenin and potentially other signaling pathways [100]. In addition, (P)RR might be proteolytically shed from membrane surface after cleavage by furin or/and ADAM19 and released into circulation as a 28-kDa soluble protein [118,119]. Plasma levels of soluble (P)RR might reflect renal damage [72,120] and/or the degree of HFrEF [121].

Prorenin/renin secretion is under the control of Ang II through a negative feedback loop. Thus, the magnitude of compensatory prorenin/renin secretion appears dependent on the degree of RAAS activation through the Ang I–Ang II arm. Hence, Ang II generation blockade with ACE-inhibitors and complete blockade of renin activity with DRIs increase the prorenin/renin plasma levels and could potentially have adverse effects through stimulation of the (P)RR-related cascades. This possibility supports the potential of (P)RR blockers as an alternative therapy for DCM-HF and other cardiovascular disorders [72,99]. However, it is unclear whether increased prorenin plasma levels result in harmful effects mediated through interaction with the (P)RR [122]. Moreover, the potential of (P)RR blockers is compromised by studies utilizing conditional knockout mouse models of (P)RR, which have demonstrated an essential RAAS-unrelated role for (P)RR in maintaining cellular homeostasis. Specific deletion of (P)RR in podocytes or cardiomyocytes resulted in the rapid onset of organ failure and subsequently animal mortality, suggesting that it is critical for heart and kidney physiology [100]. The nephron-specific knockout of (P)RR resulted in impaired V-ATPase activity and distal renal tubular acidosis in mice but did not affect RAAS [123]. 

In addition to the interaction with (P)RR, plasma prorenin/renin might directly contribute to cardiac remodeling and DCM-HF progression by non-enzymatically binding and activating the insulin-like growth factor II/mannose-6-phosphate (IGFII/M6P) receptor, which is highly expressed by cardiomyocytes and cardiac fibroblasts [75,109,124,125,126,127]. The actions of prorenin/renin through other still unknown receptors cannot be excluded [103].

It has been reported that neonatal cardiomyocytes as well as cardiac fibroblasts are able to bind and internalize recombinant renin and prorenin through IGFII/M6P receptors [109]. The IGFII/M6P receptor is elevated, and its cardiac expression plays an important role in hypertension-induced HF in spontaneous hypertensive rats. In cultured cardiomyoblast H9c2 cells and in left ventricles of hearts excised from a hypertensive Sprague Dawley rat model with abdominal aorta ligation, IGFII/M6P receptor expression was elevated in response to Ang II-induced apoptosis [128,129,130,131]. Prorenin/renin participates in structural remodeling of cardiomyocytes and demonstrate anti-hypertrophic properties [75] via their interaction with the IFGII/M6P receptor. In this way, the drugs modulating prorenin/renin levels potentially counter Ang II-dependent adverse remodeling by directing renin to act non-enzymatically through IGFII/ M6P receptors. In rat myocardial ischemia-reperfusion models, there is hormonal downregulation of cardiac IGFII/M6P receptor mRNA expression during the acute postinfarction phase [75]. In contrast, cardiac IGFII/M6P receptor expression has been shown to be increased in patients with end-stage HF [132]. Renin exerts its effect via the IGFII/M6P receptor by stimulating the elongation of cardiomyocytes [133]. This effect requires the activation of extracellular signal-regulated kinases 1/2 (ERK1/2) [134] and is antagonized by peroxisome proliferator-activated receptor gamma (PPARγ), which is known to enhance renin gene expression [135]. Thus, renin acting via IGFII/M6P receptor is the first modulator that affects cardiomyocyte length instead of cell thickness, unlike other pro-hypertrophic agents [75]. The effect of renin on the length of cardiomyocytes is attenuated in the presence of M6P, which competes with renin for the IGFII/M6P receptor [135]. Complete M6P/IGF2R knockout results in fetal overgrowth and neonatal lethality. However, the inducible cardiac muscle, skeletal muscle, and liver-specific knockouts of the IGFII/M6P receptor are not lethal and have no obvious phenotype [136]. However, renin expression level was not analyzed in these mice.

Summarizing the above, circulating prorenin/renin has the potential to directly and/or indirectly modulate HF progression (Figure 1). Direct modulation is believed to occur through stimulation of tissue-specific (P)RR and/or IGFII/M6P receptors, which are not solely specific for renin, or other still unknown receptor(s). The potential effect of direct genetic and pharmacological alterations of (P)RR as modulators of HF outcomes have been discussed elsewhere [72,75,99]. Indirect modulation of HF occurs through the enzymatic function of active renin and its downstream effects on Ang II and Ang (1–7) production, both in circulation and at the local tissue levels. Imbalance of the two pathways contributes to DCM-HF progression. A pharmacological alteration of renin activity might be beneficial in HF management [99] and results in simultaneous changes of Ang II/Ang (1–7) [137], (P)RR, and IGFII/M6P networks; the interplay of these effects remains to be investigated. We will focus on DRIs that were primarily designed to block enzymatic activity of renin. Beta-blockers and potentially vitamin D receptor activators, including vitamin D, also lower renin activity and potentially benefit HF management; however, these modulators are beyond the scope of this review [138,139,140]. To improve the outcomes of the renin activity-targeted pharmacological approaches aiming to delay or prevent DCM-HF progression, at least three major questions remain to be answered. First, does a pathological increase of renin activity in circulation begin in the presymptomatic stages (A and B) of HFrEF? Second, does an increase in plasma renin activity have a causative effect on DCM-HF progression? Third, does a precise alteration (i.e., normalization) of renin activity in plasma versus a complete suppression of renin activity have differential effects on the progression of HF?

## 3. Renin Activity as a Diagnostic and Prognostic Marker

Plasma renin activity (PRA) and active renin concentration (ARC) longitudinally increase with HF stages in experimental HF to become pathologically elevated in symptomatic HF; levels of each vary between subsets of HF patients with reduced ejection fraction (HFrEF) [15,16,141,142,143,144,145]. Still, the diagnostic and prognostic value of renin enzymatic activity as a predictor and/or modulator of presymptomatic and symptomatic HFrEF, as well as its value for guiding therapy in the absence or presence of a RAAS blockade, remains to be established. 

### 3.1. Assays of Plasma Renin Activity

Experimental studies of renin activity in patients with HFrEF are challenging; patients are heterogeneous, often receive treatments that may modify renin activity, and may have additional comorbidities which differentially affect experimental interventions during clinical trials. For example, therapies that modulate Ang II/aldosterone production and activity affect prorenin/renin levels, renin activity levels, and the potential effects of renin-targeted treatments.

**Figure 2:**
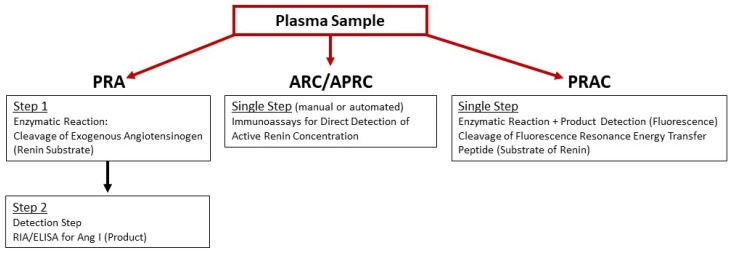
Schematic presentation of assay principles used to measure enzymatic renin activity in plasma samples: Plasma renin activity (PRA); active renin concentration (ARC)/active plasma renin concentration (APRC); and plasma renin activity concentration (PRAC).

Although circadian rhythm does not affect PRA levels [146], physical activity might. Thus, patients are recommended to sit 10–15 min prior to blood collection [82]. Precautions should be taken during blood sample collection, storage, and handling over assay performance to prevent in vitro proteolysis of prorenin/renin and the non-proteolytic conversion of prorenin to active renin by cryoactivation and/or low pH. Cryoactivation is the process of irreversible conversion of prorenin to active renin in cooled, unfrozen plasma due to unfolding of the prorenin prosegment following by its cleavage by plasma proteases. Considering that the concentration of prorenin in plasma is more than 10-folds greater than active renin, even a small modulation of the prorenin/active renin ratio in vitro might significantly compromise the final measurements [147,148,149]. Importantly, techniques and assay protocols used to measure renin activity differ between HFrEF clinical studies (Figure 2), which may impact major conclusions. Reference ranges for renin plasma activity vary; they depend on assay method and differ between commercially available kits. Reference ranges for PRA and ARC have been summarized and published [150].

#### 3.1.1. Ang I Generation Assay

Traditionally in clinical studies, renin enzymatic activity, abbreviated as PRA, is assayed by a 2-step process measuring the potential of a patient’s plasma sample to convert exogenous angiotensinogen (substrate) to Ang I (product) during in vitro incubation, followed by measurement by radioimmunoassay (RIA) or enzyme-linked immunosorbent assay (ELISA) of the generated Ang I. However, the Ang I generation step is time-consuming and time-sensitive. Depending on active renin levels in plasma, this step might take from 3 to 18 h [147,151]. Moreover, PRA measurements could be altered by the amount of endogenous angiotensinogen level in plasma samples.

#### 3.1.2. Immunoassay for Active Renin

The second method for estimation of enzymatically active renin in plasma samples is angiotensinogen-independent and technically more convenient. This assay measures an active renin concentration, abbreviated as ARC/APRC, using an antibody that is specifically directed against the active site of renin and. therefore, capable to distinguish active renin from inactive prorenin [99,151,152]. In general, these assays use principles of sandwich immunoassay; monoclonal capture antibody recognizes both active renin and prorenin, followed by a detection step with a labeled monoclonal antibody toward active renin. Several detection systems have been developed and are comprised of either manual or automated immunoassays for the quantification of ARC/APRC [152,153,154,155,156].

#### 3.1.3. Renin-Specific Substrate Cleavage Assay

An alternative strategy for renin enzymatic activity quantification utilizes exogenous fluorescence resonance transfer (FRET) peptide substrates of renin based on the octa- to deca-peptides derived from the N-terminal sequence of angiotensinogen, labeled with quencher/fluorophore pairs—DABCYL/EDANS [157,158], Dnp/Amp, or QXL™520/5-FAM (SensoLyte 390 or SensoLyte 520 assay kit, AnaSpec, Fremont, CA, USA). Cleavage of the FRET substrate results in the recovery of quenched fluorescence that can be directly monitored and conveniently quantified. Results of this assay are expressed as active enzyme concentration in nM or ng/mL. The QXL™520/5-FAM pair is superior to the other two: 5-FAM fluorophore has higher brightness and stability, and its fluorescence can be monitored with minimal autofluorescence background of test compound, cell components. and plasma proteins (SensoLyte 520 assay kit, AnaSpec, Fremont, CA, USA). The FRET-QXL™520/5-FAM peptide substrate was optimized by the AnaSpec, Inc. for human, mouse, or rat renin. Specificity of substrate cleavage was validated with several known renin inhibitors. Commercially available kits containing FRET-QXL™520/5-FAM substrates are designed for in vitro screening of renin inhibitors, are marked “for research use only”, and are not validated by the company to measure renin enzymatic activity in plasma. Hence, normal values and ranges for renin are not established for this assay. and clinical comparisons with PRA and ARC assays have not yet been performed. Although nonspecific cleavage of this FRET substrate in plasma could not be excluded, a supplementation of plasma samples with a combination of ethylenediaminetetraacetic acid (EDTA) and serine protease inhibitors phenylmethylsulfonyl fluoride (PMSF) or aprotinin chelates zinc/calcium ions required for activity of ACE and angiotensinase A and inactivates chymases and angiotensinase B. FRET-QXL™520/5-FAM containing kits were successfully adopted by several laboratories for the direct measurement of plasma renin enzymatic activity [145,159,160,161,162,163,164,165]. Importantly, the assay detected changes in plasma renin activity concentration relative to pathophysiological stresses associated with renal autograft ischemia-reperfusion injury [159], response to ARBs in normal and diabetic C57BL/6 mice [163], and chronic suppression of plasma renin activity in experimental mouse model of HFrEF with DRI aliskiren [165]. We abbreviate this assay as PRAC (plasma renin activity concentration) in order to distinguish from PRA and ARC/APRC. 

### 3.2. Prognostic Value of Plasma Renin Activity in HFrEF

Over the past decade, the prognostic value of PRA in HF has been extensively evaluated and reported. PRA was reported to be an independent prognostic marker in prospectively enrolled patients with HFrEF (*n* = 996), irrespective of medical treatment, which was additive to N-terminal-pro hormone B-type natriuretic peptide (NT-proBNP) levels and ejection fraction (EF) [166]. The independent prognostic value of PRA was reported for chronic HF patients with chronic kidney disease comorbidity. PRA in combination with NT-proBNP plasma levels identified a subgroup of high risk patients, who might benefit from more intensive care [167]. Higher PRA levels were associated with a greater likelihood for prevalence of congestive HF in a large diverse cross sectional study on hypertensive individuals [168]. Elevated PRA levels demonstrated increased risk for congestive HF and a trend toward higher mortality among patients with systolic blood pressure (SBP) ≥ 140 mmHg, but this was not true for individuals with SBP < 140 mmHg [169]. PRA was significantly elevated in ambulatory chronic HFrEF patients and in acute HFrEF patients [170]. All trials described above contain patients with concurrent HF medications (ACE-I, ARB, ARNi, etc.). The Studies of Left Ventricular Dysfunction (SOLVD) trial showed groups (control vs. HFrEF) could be stratified based on elevated PRA levels without prior exposure to ACE inhibitors but did not exclude diuretics [16]. Similarly, others reported that HFrEF patients on diuretics were more likely to have elevated PRA [171]. However, the results from Val-HeFT trials report that PRA remains a prognostic marker even in the presence of ACE inhibitors, which are known to increase PRA levels [143]. ARC was reported to be superior to PRA for the evaluation of HF severity and for independently predicting survival in HF patients who were hospitalized for management of HFrEF and were already on ACE inhibitor or ARB medications [153]. Most recently, ARC was found to be a potential biomarker for HFrEF, which had value in addition to NT-proBNP and NYHA classification, to subclassify HFrEF patients receiving RAAS blockers into HFrEF phenotypes that required adaptive therapeutic interventions [156]. 

Although differences between PRA and ARC/APRC are not clearly established in HF, specific measures of plasma renin activity may be useful for identifying individuals for whom titrated doses of renin inhibitors may attenuate the progression of HFrEF.

**Figure 3:**
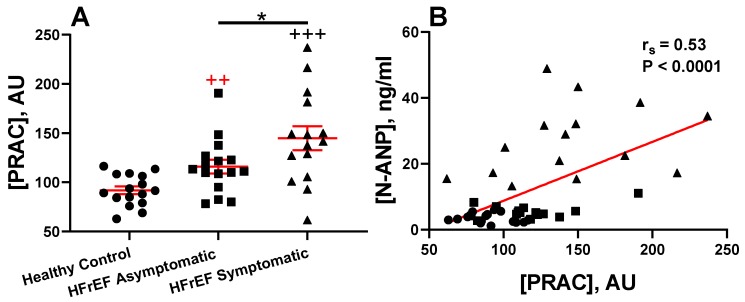
Plasma renin activity concentration (PRAC) in healthy control and heart failure (HF) patients with systolic dysfunction: (**A**) Plasma samples of healthy control patients (normal ejection fraction, EF) and patients with reduced (rEF) with and without symptomatic HFrEF. (**B**) Spearman correlation of PRAC to plasma N-terminal pro-atrial natriuretic peptide (N-ANP). All patients were males and 50–70 years old. Groups were healthy control subjects (*n* = 16), HF with reduced ejection fraction (HFrEF) asymptomatic (*n* = 16), and HFrEF symptomatic (*n* = 15). Venous blood samples were collected using EDTA-aprotinin tubes. This investigation was a part of our previously reported study [25]. This study was approved by the Institutional Review Board, and all subjects gave their informed consent for inclusion before they participated in this study [25]. Data represent mean ± SEM. ++ *p* < 0.01, (red, Control vs. Asymptomatic) +++ *p* < 0.0001 (black, Control vs. Symptomatic), * *p* < 0.05 (Asymptomatic vs. Symptomatic HFrEF). AU = arbitrary units. Comparisons between groups were calculated using the Mann–Whitney test. Statistical analysis was performed with GraphPad Prism 8.0.2 (GraphPad Software, San Diego, CA, USA). *p* > 0.05 was considered significant.

Our unpublished pilot data show that a pathological elevation of PRAC precedes the development of edema (symptomatic HFrEF) in a subset of patients with reduced systolic function with or without symptomatic HF (Figure 3A). There was no significant difference in medical management between rEF groups with or without symptomatic HF; an equal percentage of patients received beta-blockers, ACE inhibitors, or ARBs [25,82]. Patients with a significant increase in PRAC levels compared to healthy controls might benefit from the addition of DRI to standard HF therapy. Patients in this study were characterized by enzymatic downregulation of the NP system, with elevated plasma levels of NEP, atrial natriuretic peptide (ANP), B-type natriuretic peptide (BNP), and cGMP and reduced plasma levels of the pro-natriuretic peptide convertase, corin [25]. There was a positive correlation between PRAC and plasma N-terminal-pro atrial natriuretic peptide (N-ANP) (Figure 3B).

### 3.3. Renin Activity in Idopathic and Experimental (Genetic or Induced) Animal HFrEF Studies

The diagnostic value of PRA is evident in canine HF associated with idiopathic DCM (HFrEF) [172]. DCM is one of the most common heart diseases in dogs and carries a poor prognosis; it is characterized by atrial/ventricular dilatation and myocardial systolic/diastolic dysfunction [173,174,175]. Similar to humans, dogs with DCM often progress to symptomatic HF characterized by dyspnea caused by pulmonary edema and/or pleural effusion and by abdominal distention caused by ascites [172,174,176,177,178,179]. Dogs with DCM may have cough, depression, weight loss, panting, syncope, and polydipsia. Similar to humans, plasma NT-proANP levels are elevated in dogs with symptomatic HFrEF but not in dogs with non-symptomatic DCM [174]. PRA and plasma aldosterone levels were reported to be significantly increased above normal levels in dogs with symptomatic HFrEF [15,142,174]. PRA but not aldosterone levels were elevated in a subset of dogs with asymptomatic HFrEF [142].

DCM and symptomatic HFrEF have been reported by echocardiography and histological studies of nonhuman primates. However, PRA and ANP/BNP or levels for other HF markers were not reported [180,181,182,183,184].

Translational mouse and rat models of DCM-HF are invaluable experimental tools for probing single-variable research questions in a mammalian living system, without other confounders associated with clinical studies [185,186]. Fortunately, the key features of the prorenin/renin network are rather similar in humans and animals [74]. Still, the number of renin-producing kidney cells, the rate of intracellular prorenin processing, the rate of prorenin/renin activation and secretion, and the rate of angiotensinogen cleavage by active renin are species-specific [74,187]. Unlike other mammalian species, mice express one (Ren-1, ortholog of human Ren gene) or two renin genes (Ren-1 and Ren-2, tandem duplication of Ren-1, encodes non-glycosylated form), depending on the strain [188]. Although plasma renin activity and aldosterone levels showed no significant differences between one renin and two renin gene mice, C57Bl/6, C3H, and BALB/c mouse strains, which do not harbor the second renin gene (Ren-2), are preferential for translationally relevant experimental DCM-HF studies as they more closely represent the human condition [81,189,190]. Several renin knockout and transgenic animal models have been generated and described. Unexpectedly, renin null rats (Ren-1 -/-) were viable and showed a profound decrease in blood pressure and abnormal renal morphology with decreased glomeruli numbers and impaired renal function [191]. Mice lacking the Ren-1d gene display reduced plasma active renin levels, an increase in prorenin levels, abnormal renal morphology, and a sexual dimorphic hypotension in females [192]. Ren-2 gene is functionally different and may not complement the effects of Ren-1d gene in these mice. Mice lacking Ren-2 were viable and healthy without any abnormality in kidney morphology [193]. To overcome potential translational discrepancies between rodents and humans, transgenic humanized models overexpressing both human renin and human angiotensinogen have been developed in several laboratories [194,195,196,197,198,199]. However, since these models have a hypertensive phenotype, which alters HF progression, their value for DCM-HF studies is limited. 

Experimental animal studies support the diagnostic value of plasma renin activity (PRA) in symptomatic HF and HFrEF progression. PRA levels were elevated more than six-fold in rapid atrial pacing-induced symptomatic HFrEF in pigs and were resistant to treatment with ACE inhibitors, AT1 blockers, or a combination of both [200]. PRA levels were about four-folds elevated in a sheep HF model induced by rapid ventricular pacing [201]. In both models, PRA elevation was accompanied by a several-fold elevation in plasma levels of norepinephrine, epinephrine, and aldosterone. PRA levels were also elevated in dogs with advanced HF-induced rapid ventricular pacing [202]. In a drug-naïve, genetic model of HF in male rats (SHHF/Mcc-facp), PRA, ANP, and aldosterone plasma levels progressively increased with age, dilation of cardiac chambers, and development of overt HF. In this model, hypertension, PRA, and male sex were independent factors contributing to cardiac hypertrophy and HF. In female rats, the progression of PRA elevation and HF was delayed in comparison with males [203]. PRA and aldosterone plasma levels were pathologically elevated in a genetic mouse model of progressive cardiac dysfunction, leading to lethal arrhythmias due to a cardiac-specific, dominant negative form of neuron restrictive silencer factor (dnNRSF-Tg) [204]. PRA was significantly elevated in a transgenic (CSQ-tg) HF mouse model of progressive DCM, which was characterized by abnormal calcium levels, hypertrophy, cardiac fibrosis, and poor contractility and resulted in overt HF and death [205,206,207].

**Figure 4:**
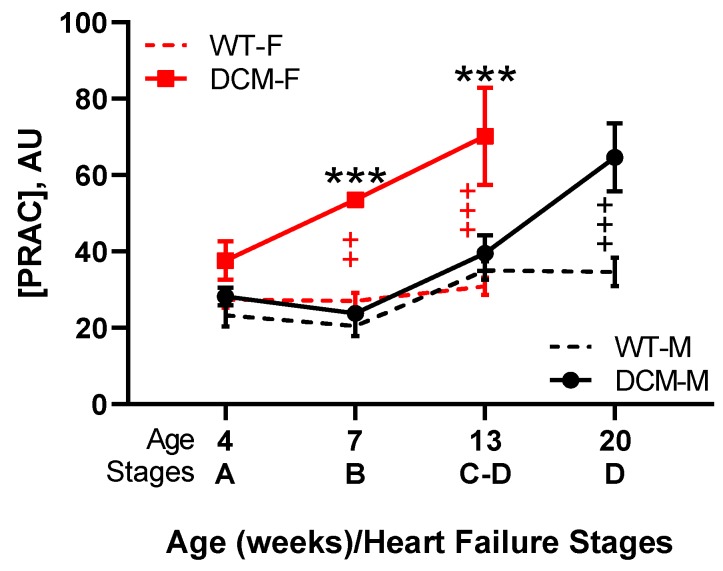
PRAC increases in a sex-dependent manner throughout the course of HFrEF progression in male and female mice with dilated cardiomyopathy (DCM). This figure combines published and unpublished data from our previously reported study [145]. Wild type males (WT-M): *n* = 5–8/age group; WT females (WT-F): *n* = 6–7/age group; DCM males (DCM-M): *n* = 7–8/age group; DCM females (DCM-F): *n* = 6–8/age group. +/red—Difference between WT and DCM females: ++ *p* < 0.01, +++ *p* < 0.001; +/black—Difference between WT and DCM males: +++ *p* < 0.001; */black—Difference between DCM females and DCM males: *** *p* < 0.001. Time-dependent differences between sexes (male vs. female) and differences between genotypes (WT vs. DCM) were analyzed by two-way ANOVA with the Bonferroni posttest correction using GraphPad Prism 8.0.2 (GraphPad Software, San Diego, CA, USA). Data are expressed as mean ± SEM. All animal study activities were approved [145].

We recently reported that PRAC levels are elevated prior to edema formation and reflect HFrEF progression in female mice with DCM due to a transgenic, dominant-negative CREB transcription factor [18,145,164,208,209]. Similar to humans, these DCM mice pass through all four Stages A–D of HF in a sex-related manner from Stage A (risk without abnormalities) to progressively declining contractile function and increasing heart dilation (Stage B) to the development of peripheral and pulmonary edema and pleural effusions with increases in plasma ANP and BNP (Stage C) to the onset of severe HF and death (Stage D) [145,210]. Pathological PRAC, plasma Ang II, and aldosterone levels increased with the progression of systolic dysfunction, cardiac remodeling, edema, and stages of HF in a sex-related fashion (Figure 4). Despite elevated PRAC levels, systolic and diastolic blood pressure were not elevated and kidney function (plasma BUN and creatinine) was within normal limits [145]. In female mice with DCM, the pathological rise in PRAC levels preceded the development of edema (Stage C) and likely contributed to the early demise of female verses male DCM mice (median survival 13.8 verses 20 weeks). Although the RAAS may be affected by sex [211,212], ovariectomy did not influence PRAC, systolic function, lung water retention, and survival [145]. Potentially, the increased PRAC levels in female mice with DCM may be driven by sex-related differences in upstream pathways related to the kinin–kallikrein/Factor XII network, which may regulate enzymatic prorenin activation [92,93].

## 4. Renin Activity as a Bio-Target for Pharmacological Intervention Using Direct Renin Inhibitors (DRI) in HFrEF

The pharmacological class of direct renin inhibitors (DRI), which are used predominantly to treat hypertension, work through high-efficiency binding to active renin [213]. As the initial step in the RAAS pathway, direct blockage of the enzymatic activity of renin has the theoretical benefit of modulating downstream molecules and receptors. In both human and animal trials, aliskiren continues to be the most frequently studied DRI; however, newer drugs are in development, such as ACT-178882/MK-1597 [214] and TAK-272 (imarikiren) [207,215] as monotherapies or in combination with other RAAS inhibitors.

### 4.1. In Clinical HFrEF

First and second generation DRIs failed due to their short durations of action, low oral bioavailability, and poor efficacy [216]. In 2007, aliskiren, a third generation orally active DRI was approved by the U.S. Food and Drug Administration for the treatment of hypertension [216]. Soon thereafter, this small-molecule inhibitor was enrolled in several clinical trials [40,41,217,218,219] to investigate the clinical effect of the drug against and in combination with standard therapeutic medications for the treatment of HF. The clinical trials were largely neutral [40,41,42,218,219,220], with most reporting no reduction in cardiovascular deaths or rehospitalizations due to DRI or the combination of RAAS blockers (ACE-I, beta-blockers, etc.). Some trials even stopped prematurely due to increases in adverse events compared to control groups. A retrospective critical evaluation of these clinical trials revealed inconsistent enrollment criteria which included HF with reduced ejection fraction (HFrEF) ranging from a ≤35% to 45% variation in the initial HF stage based on the New York Heart Association (NYHA) classification and mixed comorbidities. Only the Additive Renin Inhibition with Aliskiren on Renal Blood Flow and Neurohormonal Activation in Patients with Chronic Heart Failure and Renal Dysfunction (ARIANA-CHF-RD) trial enrolled patients with mild to severely increased plasma renin activity (PRA) at baseline [219]; aliskiren successfully lowered PRA in the treatment group after 26 weeks compared to placebo controls. However, it is important to note that there was no classification between the outcomes of trial groups based on PRA range at enrollment (mild, moderate, and severe compared to the normal level) that have been reported. One explanation for this is that standard HF therapeutics (ACE-I, ARB, beta-blockers, diuretics, etc.) increase plasma renin activity levels [82,221]; thus, the utility of these values require additional research. Also, females were significantly underrepresented, with only 15–23% of the patient population. A sub-study of the Six Months Efficacy and Safety of Aliskiren Therapy on Top of Standard Therapy, on Morbidity and Mortality in Patients with Acute Decompensated Heart Failure (ASTRONAUT) trial revealed that aliskiren significantly reduced PRA levels in a one-year follow-up. Another major conclusion was that aliskiren treatment had a greater effect on PRA, more so than plasma aldosterone modulation, and lowered NT-proBNP levels throughout treatment. PRA was also found to correlate with neurohormonal status as a prognostic and stable biomarker, with elevated PRA corresponding to the highest risk group [222]. Considering these reviews, it is possible that a therapeutic advantage of aliskiren toward HF was overlooked due to improper identification of the patient subpopulations and the limitations of clinical PRA testing versus ARC or PRAC as discussed earlier. Alternatively, these trials may have failed because they were not biomarker-guided to treat patients with increased PRA or because they were initiated when HF was too advanced (HF Stages C and D). Another explanation is that patients might not have received benefits from DRI because they already were on maximally tolerated doses of standard ACE inhibitors and ARBs [219,223].

Studies with DRI revealed that renin aggravates HF indirectly though generation of Ang I and activation of Ang I–Ang II–ATI axis or directly via renin receptors. The DRI, aliskiren has a dose-related effect on blood pressure modulation in patients [224,225]. It has been proven safe in normotensive patients from 40–640 mg with daily oral administration [226]. Most studies utilize a loading protocol with initiation at 150 mg for 1–2 weeks, followed by an increase to 300 mg daily dose per person. If not tolerated due to side effects or hypotension, reduction to 150 mg maintenance is standard. Aliskiren was shown to decrease Ang II levels in people starting at a single dose as low as 40 mg and can suppress levels by 75–89% after one week of daily administration [227]. Results from the Aliskiren Observation of Heart Failure Treatment (ALOFT) trial using the lower common dose of aliskiren (150 mg/daily) showed positive results when used in combination with standard therapies. Aliskiren was well-tolerated when administered in combination with ACE inhibitors, beta-blockers, or MRAs. Importantly, blood pressure was not modulated by aliskiren treatment alone when compared to the placebo [228,229].

Increased plasma renin activity enhances aldosterone production. The degrees of increase in plasma renin concentrations and plasma aldosterone levels correlate with prognosis in patients with HF [11,16,230]. Aldosterone has an avid, renal sodium–water retaining effect and is considered a key hormone in the development of HF through its pleiotropic actions mediated by mineralocorticoid receptors [51]. Aldosterone has adverse effects on the cardiovascular system, including prevention of myocardial neuronal reuptake of norepinephrine (thereby enhancing sympathetic drive) and potentiation of fluid overload and electrolyte imbalance [9,51,231,232]. Clinical trials utilizing mineralocorticoid receptor antagonists (MRA) with survival benefits support the concept that aldosterone activation is deleterious in symptomatic HFrEF (Stages C and D) [31]. MRAs reduce the harmful effects of hyper-aldosteronism on the cardiovascular system and have a potassium-sparing diuretic effect.

Although the majority of DRI studies have focused on patients with rEF for enrollment criteria [41,42,217,218,219,228], newer trials are starting to consider targeting renin activity in patients with preserved EF [233]. Additionally, the systemic nature, growing comorbidities and classification of HF need to be considered when attempting to modulate RAAS/NP in HF [38,39,234,235,236,237]. This review is limited to HFrEF; however, further investigation encompassing the additional causes of HF should be considered.

### 4.2. In Animal Models of HFrEF

There are growing numbers of experimental studies addressing the effect of renin activity inhibition in animal models of cardiomyopathy leading to symptomatic HF. These studies assess the outcomes of interventions that targeted renin activity solely or versus dual renin system interventions. In myocardial infarction (MI)-induced HF models, comparative treatments with ACE inhibitor, aliskiren, or a combination of both revealed that the synergism of the drug combination was more beneficial than when either agent was used alone [238]. In an induced, diabetic cardiomyopathy mouse model, aliskiren prevented cardiac systolic and diastolic dysfunctions similar to ARBs and ACE inhibitors, significantly lowered circulating pro-inflammatory cytokine levels, and protected against oxidative stress [239]. In a mouse model of acute MI, aliskiren treatment improved systolic dysfunction and prevented remodeling, apoptosis, and hypertrophy of the left ventricle [240]. Similar effects were seen in a doxorubicin-induced cardiomyopathy rat model [241]. In a randomized, blinded experimental study we found, that biomarker-guided treatment with aliskiren [145,210], in which PRAC levels were elevated at presymptomatic Stage B HF, reduced the progression of cardiomyopathy, delayed the development of edema, and significantly prolonged life [165]. 

Several experimental studies suggest that aliskiren might work not only through Ang II but also alternatively through the Ang (1–7) arm. In hypertensive rats, 50 mg/kg/day subcutaneous aliskiren tended to reduce plasma Ang II and significantly reduced plasma Ang (1–7) levels compared to vehicles [242]. In another study, aliskiren (50 mg/kg/day) upregulated the expression of AT1 and MAS receptors and downregulated the expression of AT2 receptor in rats with diabetic nephropathy [243]. Modulation of Ang (1–7) by aliskiren was reported in patients with nondiabetic chronic kidney disease [244].

In addition to being a direct inhibitor of enzymatic renin activity, aliskiren (administrated orally at 10 mg/kg/day in drinking water) was reported to increase cardiac bradykinin and kallikrein gene expression levels in TGR(mRen-2)27 rats independent of its effects on renin inhibition [245]. A subsequent study reported that aliskiren (10 mg/kg/day) protected the rat hearts from myocardial ischemia-reperfusion injury via a B2-receptor- and AT2-receptor-mediated mechanism [246]. When aliskiren was administrated subcutaneously (SC) via osmotic mini pumps in doses that do not modulate blood pressure, its effects on local tissue’s modulations were reported. Thus, in diabetic transgenic mouse models, SC aliskiren administration resulted in improvements in albuminuria and renal fibrosis by regulating inflammation and the alteration of collagen synthesis and degradation [247]. It also improved insulin resistance and adipose tissue dysfunction in type 2 diabetic mice through an increase in insulin sensitivity, insulin secretion, and adipocyte differentiation and a reduction of oxidative stress [107]. In an Apo E-/- C57/BL6 mouse model, SC aliskiren administration results in an increased number and function of pro-angiogenic cells in mice and prevented atherosclerotic lesion formation in spleen-derived pro-angiogenic cells. The effect was independent from blood pressure through lowering (P)RR expression [248,249]. The results of these studies strongly suggest that the effect of DRI on renin activity in vivo could not be simply estimated by the changes in plasma levels of Ang I and Ang II and that PRA, ARC/APRC, or PRAC assays should be used.

Of the newer DRI drugs, only limited in vivo studies have been reported. In human-renin and human-angiotensinogen double transgenic (dTg) rats, TAK-272 was found to have a greater potency and a six-fold increase in oral bioavailability when compared to aliskiren for modulating blood pressure [215]. In a calsequestrin female transgenic (CSQ-Tg, C57BL/6 x DBA/2 hybrid) mouse model of severe symptomatic HF with high mortality, oral daily TAK-272 in a dose-dependent fashion prolonged the survival and, at the highest dose (300 mg/kg), showed a significant reduction in elevated PRA, decreased cardiac hypertrophy, a two-fold increase in left ventricular EF, decreased plasma NT-proBNP levels, and decreased lung congestion—clearly signifying cardioprotection during HF [207]. In a separate cohort of CSQ-Tg mice treated with 300 mg/kg TAK-272 for one week, there was no difference in blood pressure compared to vehicle controls [207].

## 5. Conclusions

The potential value of renin activity as a diagnostic and/or prognostic biomarker of HFrEF when assessed as PRA, ARC, or PRAC is evident in a growing number of animal and clinical studies. Still, studies evaluating renin activity as a predictor of HF in presymptomatic patients are lacking. Nevertheless, experimental animal studies strongly support the potential therapeutic benefits of precisely titrating suppression of renin activity in symptomatic HFrEF. However, clinical advancement of DRIs has slowed with the termination of the aliskiren HF trials due to side effects and poor outcomes. Clinical trial enrollment of patients with advanced HF stages (C and D) may account for some inconsistencies in evaluating renin activity levels in HFrEF patients. In addition, the dose-related effects of aliskiren in HF studies have not been well-established. Most studies have defaulted to the traditional hypertensive doses of 150–300 mg/kg/daily rather than considered the benefits of lower doses observed from the experimental studies. Currently, there are new DRIs and (P)RR inhibitors under development, which suggest that the cardiovascular field is not prepared to give up on the promising pharmacologic potential of renin activity as a bio-target to delay HF progression. However, it is important to consider that future HFrEF trials will unlikely be designed in the absence of currently recommended treatments, including other RAAS blockers. To improve both the preclinical and clinical uses of renin activity as a reliable HFrEF biomarker and bio-target, consensus should be reached over laboratory assays used in different clinical studies. It is preferable that patient plasma renin activity levels be obtained prior to initiation of RAAS blockers or diuretic therapy, as these medications may elevate levels. The optimal method of diagnosing and monitoring enzymatic renin activity levels in patient plasma samples will allow for improved outcomes through the use of individualized/precision medicine approaches in HF clinical management. Significant consideration for proper trial enrollment criteria should be paramount, as experimental studies indicate that DRIs are efficacious when applied to the proper subgroups.

## Abbreviations

HF	Heart Failure
rEF	Reduced Ejection Fraction
DCM	Dilated Cardiomyopathy
ACC	American College of Cardiology
AHA	American Heart Association
SNS	Sympathetic Nervous System
NP	Natriuretic Peptide
ANP	Atrial Natriuretic Peptide
BNP	B-Type Natriuretic Peptide
NT-proANP	N-terminal-pro ANP
NT-proBNP	N-terminal-pro hormone BNP
RAAS	Renin–Angiotensin–Aldosterone System
ACE	Angiotensin Converting Enzyme
ACE-I	Angiotensin Converting Enzyme Inhibitor
ACE2	Angiotensin Converting Enzyme 2
Ang I	Angiotensin I
Ang II	Angiotensin II
Ang (1–7)	Angiotensin (1–7)
ARB	Angiotensin II Receptor Blockers
AT1	Angiotensin II Type 1 Receptor
AT2	Angiotensin II Type 2 Receptor
Mas	G Protein-Coupled Receptor Mas
MRA	Mineralocorticoid Receptor Antagonist
ARNi	Angiotensin Receptor Neprilysin Inhibitors
DRI	Direct Renin Inhibitor
NEP	Neprilysin
JG	Juxtaglomerular
cAMP	Cyclic Adenosine Monophosphate
cGMP	Cyclic Guanosine Monophosphate
(P)RR	(Pro)Renin Receptor
MAPK	Mitogen-Regulated Protein Kinase
Erk1/2	Extracellular Signal-Regulated Kinase 1 and 2
TGF-β1	Transforming Growth Factor-β1
PAI-1	Plasminogen Activator Inhibitor Type 1
IGFII	Insulin-Like Growth Factor II
M6P	Mannose-6-Phosphate
PPARγ	Peroxisome Proliferator-Activated Receptor gamma
PRA	Plasma Renin Activity
ARC	Active Renin Concentration
APRC	Active Plasma Renin Concentration
PRAC	Plasma Renin Activity Concentration
RIA	Radioimmunoassay
ELISA	Enzyme-Linked Immunosorbent Assay
EDTA	Ethylenediaminetetraacetic Acid
PMSF	Phenylmethylsulfonyl Fluoride
AU	Arbitrary Units
SBP	Systolic Blood Pressure
SOLVD	Studies of Left Ventricular Dysfunction
NYHA	New York Heart Association
ARIANA-CHF-RD	Additive Renin Inhibition with Aliskiren on Renal Blood Flow and Neurohormonal Activation in Patients with Chronic Heart Failure and Renal Dysfunction
ASTRONAUT	Six Months Efficacy and Safety of Aliskiren Therapy on Top of Standard Therapy, on Morbidity and Mortality in Patients with Acute Decompensated Heart Failure
ALOFT	Aliskiren Observation of Heart Failure Treatment
BUN	Blood Urea Nitrogen
SEM	Standard Error of the Mean
SC	Subcutaneous
dTg	Double Transgenic
CSQ	Calsequestrin
